# Novel biaxial tensile test for studying aortic failure phenomena at a microscopic level

**DOI:** 10.1186/1475-925X-12-3

**Published:** 2013-01-11

**Authors:** Shukei Sugita, Takeo Matsumoto

**Affiliations:** 1 Center for Fostering Young and Innovative Researchers, Nagoya Institute of Technology, Gokiso-cho, Showa-ku, Nagoya, 466-8555, Japan; 2 Department of Mechanical Engineering, Graduate School of Engineering, Nagoya Institute of Technology, Gokiso-cho, Showa-ku, Nagoya, 466-8555, Japan

**Keywords:** Aortic tissue, Biaxial tensile device, Microstructure, Tearing

## Abstract

**Background:**

An aortic aneurysm is a local dilation of the aorta, which tends to expand and often results in a fatal rupture. Although larger aneurysms have a greater risk of rupture, some small aneurysms also rupture. Since the mechanism of aortic rupture is not well understood, clarification of the microstructure influencing the failure to rupture is important. Since aortic tissues are stretched biaxially *in vivo*, we developed a technique to microscopically observe the failure of an aortic rupture during biaxial stretch.

**Methods:**

A thinly sliced porcine thoracic aortic specimen was adhered to a circular frame and pushed onto a cylinder with a smaller diameter to stretch the specimen biaxially. To induce failure to rupture at the center, the specimen was thinned at the center of the hole as follows: the specimen was frozen while being compressed with metal plates having holes, which were 3 mm in diameter at their centers; the specimen was then sliced at 50-μm intervals and thawed.

**Results:**

The ratio of the thickness at the center to the peripheral area was 99.5% for uncompressed specimens. The ratio decreased with an increase in the compression ratio *ε*_c_ and was 47.3% for specimens with *ε*_c_ = 40%. All specimens could be stretched until failure to rupture. The probability for crack initiation within the cylinder was <30% and 100% for specimens with *ε*_c_ <10% and *ε*_c_ >30%, respectively. Among specimens ruptured within the cylinder, 93% of those obtained from the mid-media showed crack initiation at the thin center area.

**Conclusions:**

Aortic tissues were successfully stretched biaxially until failure, and their crack initiation points were successfully observed under a microscope. This could be a very useful and powerful method for clarifying the mechanism of aortic rupture. We are planning to use this technique for a detailed investigation of events occurring at the point of failure when the crack initiates in the aortic aneurysm wall.

## Background

A thoracic aortic aneurysm (TAA) is a local dilation that occurs in the thoracic aorta. TAA usually tends to expand gradually, resulting in a fatal rupture. The overall mortality rate due to TAA rupture is more than 90% [1]. At present, aneurysms with a diameter larger than a critical value, such as approximately 5 cm, are repaired surgically. However, there is concern that aneurysms with a diameter smaller than the critical value could also rupture [2,3]. In smaller aneurysms, the wall stress should be low, indicating weakening of the aortic wall. However, recent studies reported that ascending TAAs were not associated with wall weakening [4-6]. Thus, it is essential to investigate the rupture mechanism. Although the natural history of TAAs are not defined [7], they show reduced elastin and unaltered collagen content [5], whereas collagen increases in abdominal aortic aneurysms [8]. Therefore, simultaneous observation of the rupture phenomenon and microstructure of the aorta may provide valuable insights into rupture mechanisms.

A number of studies have used a uniaxial tensile test *in vitro* to determine mechanical properties of soft tissues. However, aortic tissues *in vivo* are stretched biaxially in longitudinal and circumferential directions due to blood pressure and axial tethering, indicating that a biaxial stretch test to be a better method for mechanical tests of aortic tissues. Elastic properties of aneurysmal tissues obtained with a biaxial tensile test have been reported [9-11]. In conventional biaxial tensile tests, the specimen is hooked with threads like a trampoline. In such a setup, cracks easily initiate from the hooked sites due to the concentration of stress at those points; thus, making it difficult to stretch specimens until rupture under such conditions. To rupture aneurysmal specimens, pressure-imposed test systems [12-14] have been developed and used successfully to determine the mechanical parameters of TAA specimens at rupture [15]. However, this system cannot be used to observe changes in the microstructure of a specimen during stretch because specimens in this test are deformed three-dimensionally, and it is therefore very difficult to continuously observe a specific point on the specimen under a microscope. Wicker et al., [16] and Chen et al., [17] examined the three-dimensional (3D) microstructure of tubular segments of healthy aortas during inflation and axial extension, although they did not apply pressure until rupture. They might have been able to observe changes in the 3D microstructure until rupture if they could have imposed rupture pressure on the specimens. However, most aneurysmal specimens obtained during surgery are in small pieces rather than whole segments. Thus, accumulation of adequate data can be very difficult with an inflation test of tubular segments of an aneurysm.

Biaxial stretch has been performed for cells on a rubber sheet by indenting the sheet that is fixed on a ring frame with a hollow cylinder under a microscope [18,19]. This method might be applicable to a biaxial tensile test until failure to rupture because the specimen is not fixed on the frame at points, but rather along a continuous line, and extreme stress concentrations associated with the use of hooks can be avoided. Furthermore, this method is ideal for stretching specimens biaxially while observing deformation under a microscope. However, it is still difficult to observe the crack initiation site in detail because (1) the position where the crack initiates is unpredictable and (2) the crack initiation point is not clearly visible for a crack that usually initiates at the rim of the cylinder where stress concentration appears.

In this study, we propose a novel technique for applying biaxial stretch to aortic tissues until failure to rupture under a microscope while controlling the crack initiation point within a desired region. We developed a biaxial tensile tester with a mechanism similar to that used for the biaxial stretching of cells [18,19]. To induce specimen failure at a desired position, we devised a novel method for thinning the specimen locally to induce stress concentration at a specific region.

## Methods

### Biaxial tensile tester under a microscope

**Figure 1:**
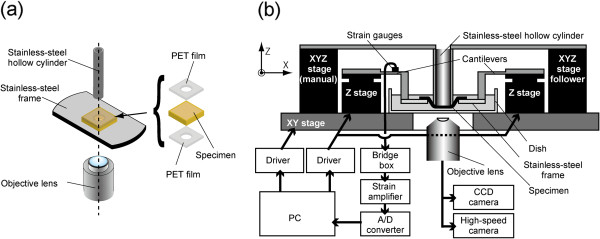
**Schematic illustrations of the experimental setup.** Mechanism for applying a biaxial stretch to a specimen under a microscope **(a)** and the entire experimental system **(b)**.

The basic mechanism of the biaxial tensile tester under a microscope was similar to that used for the biaxial stretching of cells [18,19]. Figure 1a shows a schematic illustration of the tester. The tester was designed for translucent specimens, which are 15 × 15 mm^2^. Very thin specimens were glued and sandwiched between two 20 × 20 mm^2^ polyethylene terephthalate (PET) film sheets with holes 10 mm in diameter at their centers to ease specimen handling. The specimen was then glued onto a stainless steel frame with a hole, which was 10 mm in diameter. A stainless steel hollow cylinder 6 and 8 mm in inner and outer diameters, respectively, was placed above the center of the specimen in the hole. The metal frame was then moved toward the cylinder to stretch the specimen biaxially. Figure 1b is a schematic illustration of the entire experimental system. To control its position, the cylinder was fixed on a manually operated XYZ stage (TSDS255S; Sigma Koki, Tokyo, Japan). The frame was fixed on two Z stages (SGSP80-20ZF; Sigma Koki) through cantilevers. By synchronously moving these Z stages upwards, the specimen was pushed onto the cylinder. These XYZ and Z stages were fixed on a XY stage (BIOS-225TWI; Sigma Koki) to allow observation of any part of the specimen, and the XY stage was set under an inverted microscope (IX71; Olympus, Tokyo, Japan). The Z and XY stages were controlled with software (SGTERM and Software Joystick; Sigma Koki) on a personal computer (PC). Specimen images were captured on a charge-coupled device (CCD) camera (Abrio-LS; CRi, Woburn, MA, USA) through a 2× objective lens (PLAPON2×; Olympus).

To measure the force *F* applied by the cylinder to the specimen, strain gauges were bound on the cantilevers. Data measured with the strain gauges were recorded with a data acquisition system (LabView 2010; National Instruments, Austin, TX, USA) on the PC through a bridge box (DB120A; Kyowa Electronic Instruments, Tokyo, Japan), a strain amplifier (DPM911A; Kyowa Electronic Instruments), and an analog–digital converter (NI USB-6289; National Instruments). We calibrated the load cell and found that this device could measure the force acting on specimens with a resolution of 12 mN and that the relationship between the force and the measured voltage was linear with a correlation coefficient of 0.999.

### Equibiaxial tensile test for a homogeneous and isotropic specimen

To confirm that the specimen could be stretched equibiaxially with this tester, a polydimethylsiloxane (PDMS) sheet was used as a homogeneous and isotropic specimen. PDMS prepolymer (Sylgard 184; Dow–Corning, Midland, MI, USA) was mixed with curing reagent at 10:1 (w/w), spread on a dish, and cured at 75°C for 4 h. For strain markers, black lacquer was sprayed on the surface of the approximately 50-μm thick PDMS sheet. The sheet was then cut into 15 × 15 mm squares, and a square sheet was glued on the metal frame with modified silicone adhesive (Super X; Cemedine, Tokyo, Japan). PET films were not used for this test. The specimen was then stretched biaxially with the tester. Since precise determination of the origin was important for the analysis of stress–strain curves, the height of the Z stage *h* was taken as 0 when 12 mN (0.01 V) of the force *F* was applied to the specimen. The Z stages were moved stepwise by Δ*h* = 0.5 mm while measuring the force *F* and capturing images until specimen failure. The Z stage was moved after the force *F* became stabilized; i.e., changes in the force *F* became smaller than 12 mN/min. This normally occurred in approximately 10 min, but sometimes took 50 min.

The images obtained during the biaxial tensile test were analyzed with the image analysis software ImageJ 1.42i (National Institutes of Health, Bethesda, MD, USA). The black lacquer markers located at the center of the specimen and eight arbitrary surrounding points located at almost equal intervals in a circumferential direction were selected and tracked with the particle tracking tool MTrackJ [20]. The distances between the center marker and the surrounding eight markers were measured, and the nominal strains were calculated for the eight points. We also constructed a finite element model to simulate an equibiaxial tensile test for a homogeneous and isotropic specimen. Material parameters were chosen to simulate the PDMS sheet and aortic specimen (see Additional file 1 for details).

### Preparation of aortic slices thinned at their centers

**Figure 2:**
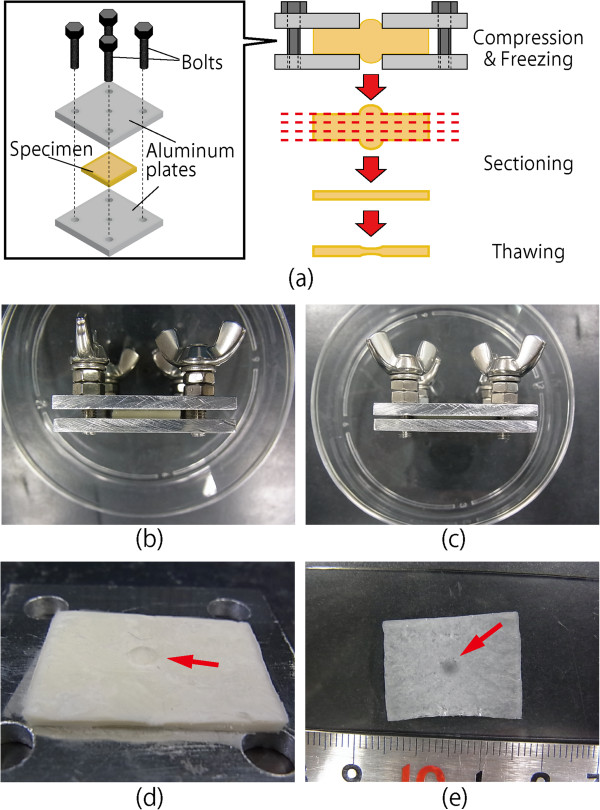
**Method for locally thinning a specimen at the center.** Schematic illustrations of the method **(a)** and images of a specimen before compression **(b)**, after 40% compression **(c)**, after freezing and removal of an aluminum plate **(d)**, and after slicing and thawing **(e)**. A boss is evident in the frozen specimen (arrow in **d**). The dark spot at the center indicates successful local thinning of the specimen (arrow in **e**).

Figure 2 shows schemata and photographs of the process used to locally thin a specimen at its center. Porcine thoracic aortas (PTAs) obtained from a local slaughterhouse were used as specimens. After loose connective tissues were removed, the PTAs were cut into rectangular specimens (15 mm in the longitudinal direction and 20 mm in the circumferential direction). These specimens were sandwiched between two metal plates having center holes 3 mm in diameter (Figure 2b). The thickness of each specimen was measured four times at different locations with a dial gauge by subtracting the thickness of the metal plates from the total thickness. The sample was then compressed 0–40% (Figure 2c) and frozen at −80°C for 10 min to fix the specimen shape. After the metal plates were removed (Figure 2d), the frozen sample was embedded in Tissu Mount (Chiba Medical, Saitama, Japan), frozen in liquid nitrogen, and sectioned with a cryostat (CM3050SIV; Leica Microsystems, Wetzlar, Germany) into 50-μm sections. The sections were obtained sequentially from the intimal to the adventitial sides of the aorta.

To measure the thickness of the specimens, some of the sample was thawed on a glass slide at room temperature (Figure 2e) and cut with a surgical knife to produce a sample of approximately 1 mm width that included the thinned area. This cut sample was mounted on a glass slide and rotated 90°, and its image was captured under the microscope. The captured image was binarized using the Otsu method [21], and the thickness was measured with ImageJ. For determining the thicknesses at the thin center area and peripheral areas, 1-mm wide regions were selected, and the average thickness was obtained for each region.

### Biaxial tensile test of aortic slices

To determine whether cracks initiated at the center of the specimens, the PTA slices prepared in the previous section were glued between two PET sheets with cyanoacrylate adhesive, and a biaxial test was performed. Z stages were elevated 0.1 mm/s while specimen images were captured with a high speed camera (Exilim EX-F1; Casio Computer, Tokyo, Japan) at 300 frames/s to determine the crack initiation site. The location where the crack initiated was divided into areas inside and outside the hollow cylinder, and the area inside the cylinder was further divided into three areas: the center (thin) area, the edge of the thin area, and other areas inside the cylinder (thick area). Biaxial tests were performed for slices obtained sequentially from the intimal to the adventitial sides, and these specimens were grouped into three categories of equal intervals in a radial direction: sub-intima, mid-media, and sub-adventitia.

## Results

### Biaxial tensile test of PDMS sheet

**Figure 3:**
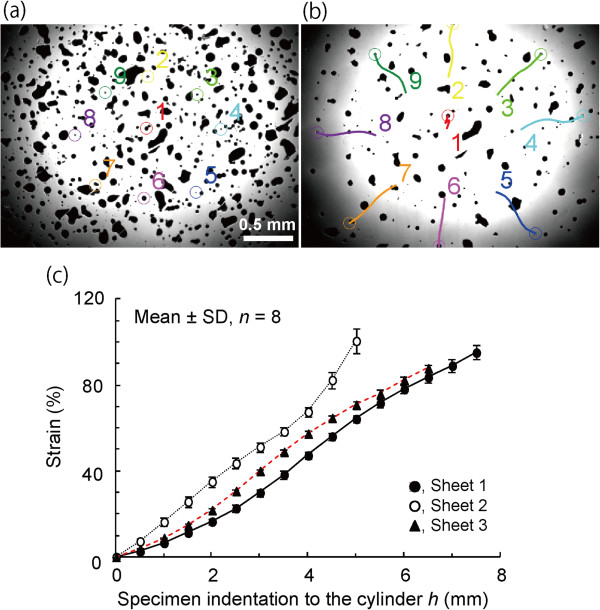
**Biaxial test of PDMS sheets.** Typical images of the sheet before **(a)** and after **(b)** biaxial stretching and changes in the strain and its standard deviation in three PDMS sheets **(c)**. Nine markers were numbered **(a)** and tracked with colored lines **(b)**. Changes in the distances from the center marker 1 were measured for the other eight markers, and their nominal strains were calculated and averaged for each sheet **(c)**.

Figure 3a and b show typical images of the PDMS sheet when the height of the Z-axis stage, i.e., the indentation of the specimen to the hollow cylinder, was *h* = 0 and *h* = 4.5, respectively. All tracked black lacquer dots moved outward in a radial direction. The strains between the center and other dots increased almost linearly with the indentation of the specimen to the cylinder, and the coefficient of variance of strains in eight approximately equiangular directions was 3.0 ± 0.9% (mean ± SD, n = 3) at *h* = 4.0 (Figure 3c). These results revealed that a homogeneous isotropic specimen could be stretched equibiaxially with this tester.

### Thickness distributions of PTA specimens thinned at their centers

**Figure 4:**
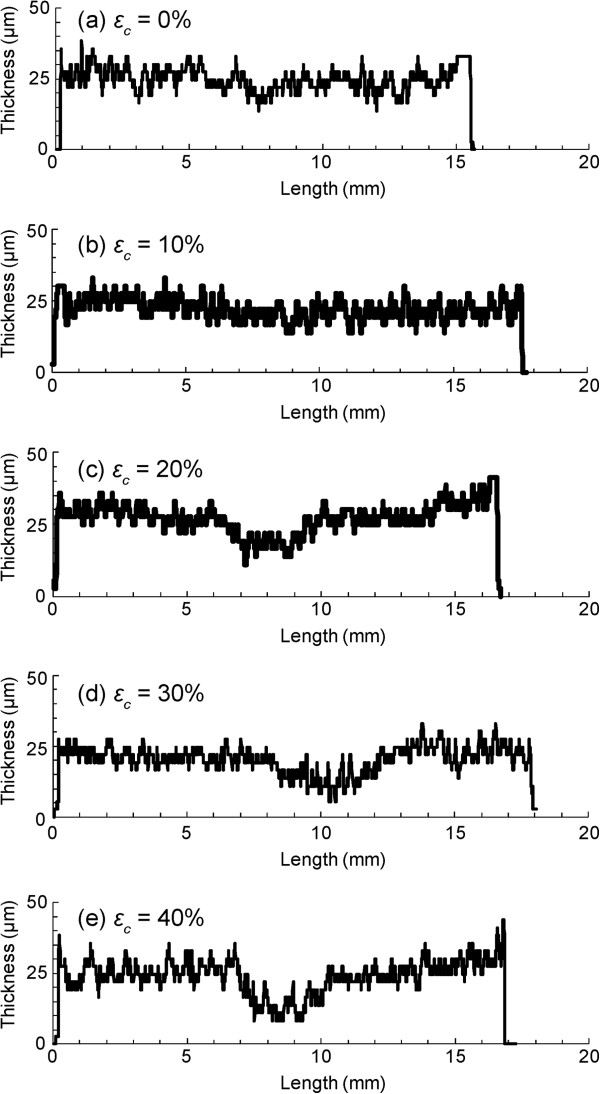
**Thickness distributions of PTA specimens at various compression ratios *ε***_***c***_**.** Local thinning around the center is evident in specimens with a compression ratio above 20%.

**Figure 5:**
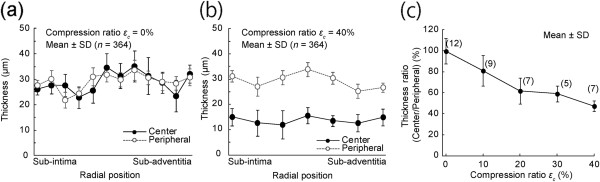
**Effects of compression ratio on the center and peripheral thicknesses of PTA slices.** Thicknesses of 12 slices obtained from various radial positions of a PTA segment are shown at a compression ratio of 0% **(a)**. Similarly, thicknesses of seven slices obtained from another PTA segment are shown at a compression ratio of 40% **(b)**. The relationship between the relative thickness at the center and the compression ratio is shown in **(c)**. In panels **(a)** and **(b)**, thicknesses were measured along a length of 1 mm (364 pixels) and averaged. Since no systematic changes were found in the radial direction, all samples obtained in various radial positions were cumulated in panel **(c)**.

Figure 4 shows typical graphs of the thicknesses of PTA specimens compressed to 0–40%. The thicknesses were nearly uniform for uncompressed specimens. There was a tendency for a higher compression ratio *ε*_c_ to result in a thinner specimen at the center area. Whereas the average thickness at the center was similar to that at the periphery for an uncompressed specimen (Figure 5a), it was significantly smaller in the center for specimens compressed by 40% (Figure 5b). The relative specimen thickness at the center was 99.5% and 47.3% for specimens compressed by *ε*_c_ = 0% and 40%, respectively (Figure 5c). The ratio decreased significantly and almost linearly with the increase in the compression ratio *ε*_c_ (*P* < 0.01, *R* = 0.97). No clear relationship was found between the radial position and thicknesses at both the center and peripheral areas (Figure 5a and b).

### Biaxial tensile test of aortic tissue

**Figure 6:**
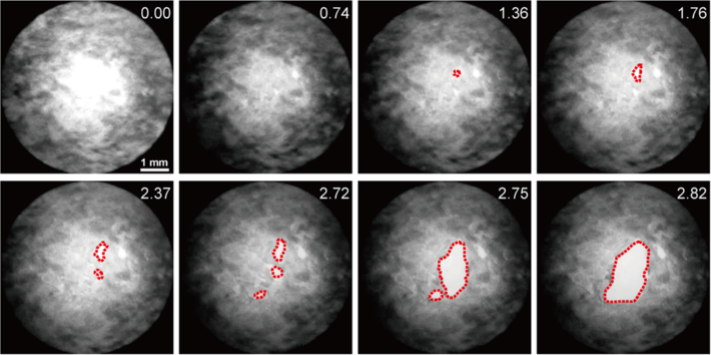
**Time-lapse images of a rupturing PTA specimen obtained from the mid-medial region.** The red dotted lines indicate the shapes of cracks. The numbers in the upper-right corners indicate specimen indentation to the cylinder *h* after force application.

**Figure 7:**
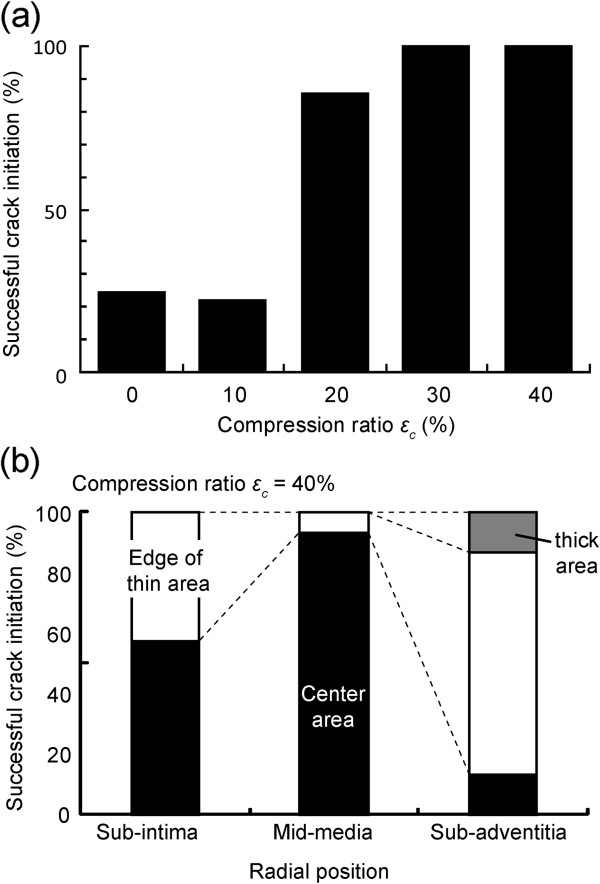
**Effects of compression ratio and radial position on the crack initiation area.** Successful crack initiation at the area inside the hollow cylinder for specimens obtained in all regions **(a)** and the change in crack initiation area with radial position for specimens compressed by 40% before freezing **(b)**. For each position, 14–15 slices were obtained from four PTA blocks and tested.

All PTA specimens could be stretched until failure by the biaxial tensile tester. The first crack initiated at various positions depending on *ε*_c_ and the radial positions. Figure 6 shows time lapse images of failure behavior of an aorta in which a crack initiated at the center area. After the crack initiated at the center area, it expanded gradually and connected with other cracks, in some but not all specimens, causing failure to rupture in the specimen. The probability of crack initiation at an area inside the hollow cylinder was 100% for specimens compressed by more than 30% (Figure 7a). In addition to the compression ratio, the location where cracks initiated depended on the radial position of the PTA. For specimens with *ε*_c_ = 40%, the probability for crack initiation at the center area was high (93%) for samples obtained from the mid-media compared with samples obtained from other radial positions (sub-intima, 53%; sub-adventitia, 13%; Figure 7b). This tendency was also found in specimens with *ε*_c_ = 30%.

## Discussion

We developed an equibiaxial tensile test system that is capable of stretching specimens until failure to rupture, while observing the microscopic deformation of the specimen. All specimens tested were successfully stretched until failure to rupture. Equibiaxial stretch was confirmed by showing that the standard deviation of strains within a homogeneous and isotropic PDMS sheet was small (3.0%). To limit crack initiation to the area inside the hollow cylinder, the center of the specimen was thinned. We confirmed that all specimens compressed by more than 30% exhibited crack initiation in the desired area. These techniques enabled us to observe the crack initiation process in detail in order to understand events occurring at the crack initiation position in the aorta. To our knowledge, this is the first report of soft tissues being stretched biaxially until failure and of the microscopic observation of the crack initiation point.

The first step for the studying aortic aneurysm failure phenomena requires knowledge at a microscopic level of normal aortic tissue failure in response to overload. If the test specimen is homogeneous like normal healthy aorta, failure phenomena observed at a thin point may well represent failure phenomena in the material. In contrast, if the specimen is heterogeneous like aneurysmal tissue, failure phenomena observed at the thin point may not well represent the failure phenomena of the tissue. In such a case, a statistical approach might be effective. By repeating this series of experiments many times, it may be possible to identify the weakest failure mode.

The present results indicated that one of the conditions necessary for causing a crack in the central area of the specimen was the ratio of the sample thickness at the center to that at the periphery. Finite element analysis showed that the von Mises stress at the area where the specimen made contact with the metal cylinder was 25% higher than the stress at the center of the specimen (see Additional file 1). The results showed that the probability of crack initiation in the central area of the specimen increased when the specimen was compressed by more than 20%. The thickness at the center was more than 25% less than that at the periphery in such specimens. Since the stress appearing in the specimen wall should be inversely proportional to its thickness, this may explain why the probability for crack initiation in the central area was high for specimens compressed by more than 20%. The compression ratio necessary to induce a crack in the central area of the specimen might be determined from the ratio of the stress at its center to that near the metal cylinder.

**Figure 8:**
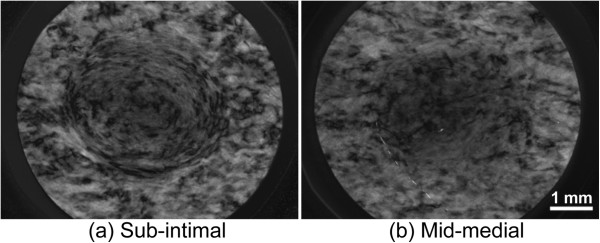
**Typical retardance images of the central area of specimens obtained from different radial positions.** Images obtained from sub-intimal **(a)** and mid-medial **(b)** specimens.

In addition to the thickness of the specimen, the radial position of the aorta affected the location of crack initiation. Most of the sub-adventitial and some of the sub-intimal specimens compressed by 40% tended to initiate cracks at the edge of the thinner area. Since the thicknesses at the thin center and the peripheral area did not change significantly among specimens obtained from various radial positions (Figure 5a and b), the shape of the specimens could not be the primary reason why cracks initiated at the edge of the central thin area. A retardance image, which shows an index of collagen density in aorta [22], may provide an explanation. Figure 8a and b show retardance images of sub-intimal and mid-medial specimens, respectively. In general, sub-intimal specimens showed a circular pattern of retardance, and collagen fibers at the thin center area appeared to show a weak connection with the collagen fibers in the periphery. This weak connection might be responsible for crack initiation at the edge of the thin central area.

Normal strain, i.e., areal strain in the case of biaxial stretch, of the PTA at rupture was 0.50 ± 0.11 (n = 23) in this study. Because the normal strain at rupture in biaxial stretch was not found, we estimated it from the result of the uniaxial tensile test that we conducted in a previous study [14]. PTA specimens were obtained without sectioning, and a uniaxial tensile test was performed in longitudinal and circumferential directions. The normal strain at failure was 0.83 ± 0.12 (mean ± SD, n = 6) for the longitudinal and 0.74 ± 0.16 (mean ± SD, n = 6) for the circumferential directions. Areal strain calculated from the smaller strain at failure in the uniaxial tensile test was approximately 0.50, which was comparable to the normal strain at failure obtained in this study.

The techniques proposed in this study may enable us to understand events occurring at the point where a crack is initiated in the aorta. Since the specimens were observed using a 2× objective lens with a bright field of view, details of the microscopic structure of the aorta were not obtained in the present study. However, by staining intramural constituents in the aorta with a labeling agent such as an antibody and using an objective lens with a higher magnification, we may be able to investigate the relationship between the distribution of intramural constituents and aortic failure. Furthermore, since we found that the collagen microstructure in the aorta could be observed using a birefringent imaging system [22], the combination of the technique outlined in this study and birefringence observation may clarify the effect of the microstructure of collagen on aortic failure.

This technique could have some limitations. First, it has been reported that freezing and thawing does not affect the mechanical properties of the aorta. Although freezing may damage cells in tissues, the stretching force applied to cells was much smaller than that applied to the extracellular matrix such as elastin and collagen. Since the effects of freezing on elastin [23] and collagen [24] are considered to be small, we believe that the influence of freezing and thawing was negligible in the present study. Second, in our study, the specimens were compressed by no more than 40% at most during sample preparation. Compression of 40% in the radial direction corresponds to 29% of the equibiaxial stretch in the circumferential and longitudinal directions due to the incompressibility of the wall material. In physiological states, 30% stretch is normal in both the circumferential and longitudinal directions. Thus, the amount of compression used in this study should not have been critical. Third, collagen fibers adjacent to the surface during sectioning may have been destroyed, which might affect mechanical properties. However, we confirmed that sectioning specimens into 100 μm did not affect the mechanical properties (unpublished data). Therefore, any influence of sectioning should have been minor. Fourth, the annular-shaped arterial wall tissue was flattened between the two metal sheets during the sample preparation. When aortic rings are cut radially, the rings generally open up due to the residual stress [25]. For PTA, the opening angle, which is the angle extended by two radii joining the midpoint of the inner wall to the tips of the ends, was reported to be approximately 50° [26], and the ratio of the thickness to the inner radius was approximately 0.1. This opened-up ring was considered to be in a zero-stress state. When the zero-stress state specimen was flattened, strains at the inner and outer radii were calculated to be no more than 5%, whereas the physiological strain is several 10%. Furthermore, when the specimen was placed on the aluminum plate to compress it, it flattened easily due to the surface tension of the saline. Therefore, since the force and strain were small when the specimen was flattened, the effect of flattening would have been very small. Moreover, the opening angle of the aneurysm tissue was empirically large, similar to that of an atherosclerotic aorta [27]. We therefore believe that the influence of flattening would not be a critical factor.

## Conclusions

We have developed an equibiaxial tensile tester for thinly sliced aortic specimens, which can be used to microscopically observe the microstructure of a specimen at the location of crack initiation. By applying this method to PTAs in which the centers of the specimens were thinned, we succeeded in observing the failure phenomena inside the thin area. The probability for crack initiation in the thin center area was higher for sections obtained from the mid-wall than for those from the sub-intimal and sub-adventitial sides. The technique developed in the present study will be useful for clarifying the mechanism of aortic rupture.

## Abbreviations

*ε*_c_: Compression ratio; TAA: Thoracic aortic aneurysm; PET: Polyethylene terephthalate; PC: Personal computer; CCD: Charge-coupled device; PDMS: Polydimethylsiloxane; PTA: Porcine thoracic aorta; SD: Standard deviation.

## Competing interests

The authors declare that they have no competing interests.

## Authors’ contributions

SS planned the study, developed the test device, carried out all of the biochemical tests, and drafted the manuscript. TM conceived the method for specimen thinning, participated in study design and coordination, and reviewed the draft of the manuscript. All authors read and approved the final manuscript.

## Supplementary Material

Additional file 1 Finite element analysis of stress in a specimen during the biaxial tensile test. Click here for file
