# Supplementary material for: Novel biaxial tensile test for studying aortic failure phenomena at a microscopic level
Source: Biomed Eng Online. 2013 Jan 11;12:3. doi: 10.1186/1475-925X-12-3 (PMC3560224; doi:10.1186/1475-925X-12-3)
Supplement: Additional file 1 — Finite element analysis of stress in a specimen during the biaxial tensile test. [file 1475-925X-12-3-S1.doc]

**Additional file 1. Finite element analysis of stress in a specimen during the biaxial tensile test**

Stress distribution in specimens during the biaxial tensile test was calculated using finite element analysis. A finite element model was constructed with 800 four-node shell elements for the metal cylinder and 3750 three-dimensional solid elements for the sample based on equibiaxial tester geometry as a quarter model (Fig. S1). For indenting the cylinder into the specimens, an imposed velocity equivalent to 0.1 mm/s indentation was applied to the metal cylinder in the Z direction, and the specimen edge bound to the metal frame was constrained in all directions. A Poisson’s ratio of 0.3 and an elastic modulus of 210 GPa were used for the mechanical parameters of the cylinder, and a Poisson’s ratio of 0.49 and an elastic modulus of 1.3 MPa were used to simulate the mechanical behavior of the PDMS sheet as well as an aortic specimen. The friction coefficient between the specimen and the cylinder varied from 0.0 to 0.5. Finite element analysis was conducted using a commercial code (RADIOSS v.10; Altair Engineering), and the distribution of von Mises stress was analyzed. The maximum stress near the cylinder *σ_cylinder_*, normalized with the value at the center point of the specimen *σ_center_*, was also calculated.

A typical result for von Mises stress in a specimen with friction coefficient of 0.0 is shown in Fig. S2. Stress in the specimen was higher at the area where the metal cylinder made contact. The ratio of the stress *σ_cylinder_*/*σ_center_* was 1.18 at friction coefficients of 0.0 to 1.62 and at the friction coefficient of 0.5. Since the friction coefficient between a metal catheter and blood vessel was reported to be approximately 0.1 [1], the ratio *σ_cylinder_*/*σ_center_* was considered to be 1.25. This ratio did not change by more than 0.5% when Poisson’s ratio of the PDMS sheet was changed from 0.485 to 0.495.

Stress at the top layer of the specimen edge was high in FEM analysis. However, we rarely observed crack initiation at that area. In the FEM analysis, the specimen edge bound to the metal frame was constrained in all directions, whereas the actual specimen was glued between two PET sheets and fixed to the metal frame with cyanoacrylate adhesive. The edges of these PET films can deform, and this might decrease the stress concentration near the edge. In this sense, the boundary condition of the specimen edge in the FEM analysis may not represent the physical conditions correctly. However, we did this simulation to reveal the stress values near the cylinder and in the center of the specimen, both of which were far from the specimen edge and do not seem to be affected by the boundary condition. Thus, we believe that the boundary condition difference between simulation and experiment did not affect the conclusion of this paper.


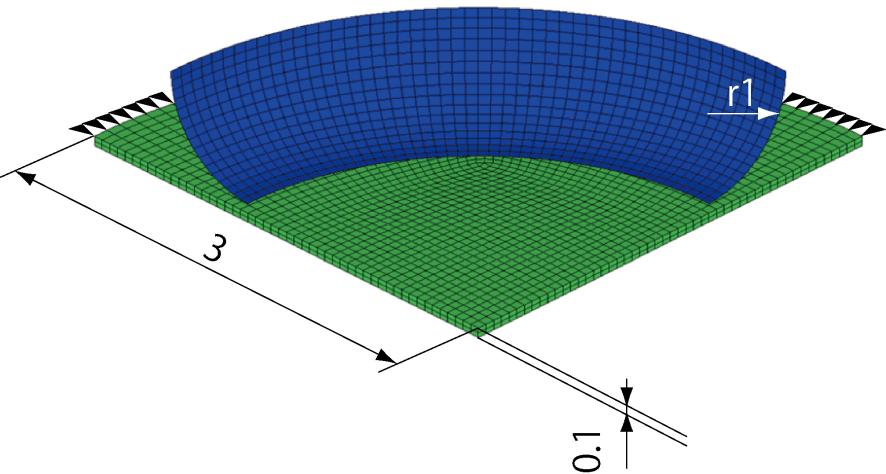


Fig. S1. Finite element model of the equibiaxial stretch tester. The metal cylinder is modeled as a shell (blue) and the specimen is modeled as a 3-D solid (green).


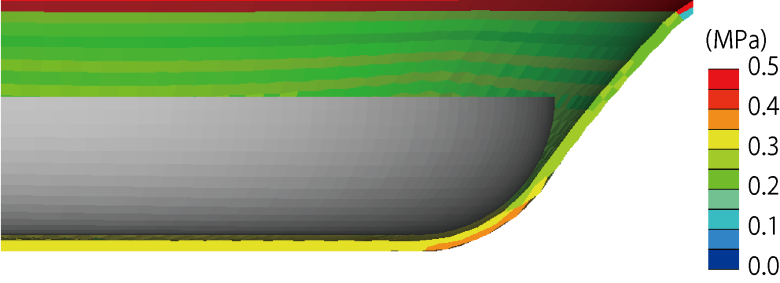


Fig. S2. Typical von Mises stress data of a specimen at a friction coefficient of 0.0. The metal cylinder is shown in gray.


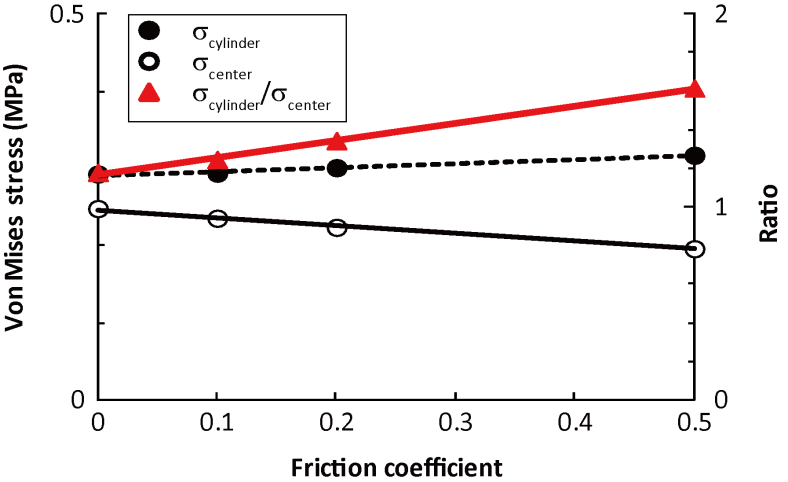


Fig. S3. Changes in von Mises stress at the center *σ_center_* and the area near the metal cylinder *σ_cylinder_* of the specimen, and their ratios with respect to the friction coefficient.

**References**

1. Takashima K, Shimomura R, Kitou T, Terada H, Yoshinaka K, Ikeuchi K**: Contact and friction between catheter and blood vesse**l*. Tribology Internationa*l 2007**, 4**0:319-328.
